# Hearing rehabilitation with Baha® transcutaneous and percutaneous systems

**DOI:** 10.1590/2317-1782/20232022271en

**Published:** 2023-10-23

**Authors:** Eliane Aparecida Techi Castiquini, Kátia de Freitas Alvarenga, Lucilena Miranda de Souza, Valdéia Vieira de Oliveira, Juliana Nogueira Chaves, Luiz Fernando Manzoni Lourençone, Rubens Vuono de Brito

**Affiliations:** 1 Divisão de Saúde Auditiva, Hospital de Reabilitação de Anomalias Craniofaciais - HRAC, Universidade de São Paulo - USP - Bauru (SP), Brasil.; 2 Departamento de Fonoaudiologia, Faculdade de Odontologia de Bauru - FOB, Universidade de São Paulo - USP - Bauru (SP), Brasil.; 3 Programa de Residência Multiprofissional em Saúde Auditiva, Hospital de Reabilitação de Anomalias Craniofaciais - HRAC, Universidade de São Paulo - USP - Bauru (SP), Brasil.; 4 Hospital de Reabilitação de Anomalias Craniofaciais - HRAC, Universidade de São Paulo - USP - Bauru (SP), Brasil.; 5 Curso de Medicina, Faculdade de Odontologia de Bauru - FOB, Universidade de São Paulo - USP - Bauru (SP), Brasil.

**Keywords:** Bone Conduction, Ear Malformation, Speech Audiometry, Hearing Loss, Hearing Aids

## Abstract

**Purpose:**

Longitudinally verify the influence of auditory tonal thresholds obtained with transcutaneous and percutaneous bone-anchored hearing aids on speech perception in individuals with external and/or middle ear malformation and chronic otitis media.

**Methods:**

Observational, retrospective, longitudinal follow-up study of 30 unilateral users of the transcutaneous and percutaneous Baha® system for the collection of secondary data on pure tone thresholds obtained through free field audiometry and sentence recognition threshold in silence and noise in conditions: without the prosthesis; at the time of activation; in the first month of use (post 1); and in the third month (post 2).

**Results:**

There was a significant difference between pure tone thresholds obtained at frequencies of 3 and 4kHz with better results for the percutaneous technique at all evaluation moments. For both systems, better performance was observed in sentence recognition in silence and in noise, with a significant difference in activation (p<0.001), but it remained stable during the other evaluation moments. The percutaneous system showed better benefit in recognizing sentences in noise only on activation (p=0.036), when compared to the transcutaneous system.

**Conclusion:**

The percutaneous system provided better audibility for high frequencies; however, such audibility did not influence sentence recognition in the silent situation for both systems. For the noise situation, better responses were observed in the percutaneous system, however, the difference was not maintained over time.

## INTRODUCTION

In the context in which it is not possible to adapt hearing aids by air conduction due to anatomical, physiological or pathological factors, such as recurrent malformations and infections of the outer and/or middle ear or allergic processes related to the earmold, the bone anchored hearing aids (BAHA), enable the auditory rehabilitation process^(1,2)^. This type of bone conduction electronic device provides the necessary audibility and speech perception for the acquisition and development of oral language, in addition to contributing to school development with the insertion of the individual in the job market and favoring socialization, with consequent impacts on the quality of life.

The indication for the BAHA is based on audiological and medical criteria, such as bone conduction hearing thresholds and skullcap thickness, respectively^(3,4)^. Individual needs such as aesthetic concerns, manual dexterity and expectation of social and professional benefits must also be considered. In this sense, the individual must be encouraged and stimulated to actively participate in the nomination process for assertive decision-making^(4)^_._

The BAHA eliminates the insertion of molds, eartips or any sound conductive material in the external auditory meatus and have two components: the external (sound processor) and the internal (implantable), being classified as transcutaneous and percutaneous. In both, the sound is transmitted as a mechanical signal through skull vibration directly to the cochlea^(4,5)^, which results in wave propagation along the basilar membrane and stimulation of the auditory nerve^(6)^.

Cochlear's BAHA, the Baha® system, was developed by Tjellstrom in 1977, using the Branemark implantation system^(7)^. In the percutaneous system (Baha® Connect), the sound processor is attached to the skull bone through an abutment (pillar), and a titanium screw with no barrier between the two components. The transcutaneous system (Baha® Attract), in turn, has, in addition to the titanium pin inserted into the bone, an internal magnet through which the processor is coupled to the external magnet that will remain in direct contact with the skin^(4,8)^. It is important to highlight that the same sound processor model is used in both systems.

In Brazil, the first surgeries with the Baha® system were performed in a public service (Hospital de Reabilitação de Anomalias Craniofaciais - University of São Paulo/Brazil) in the 90s through a research project aimed at syndromic individuals with conductive and mixed hearing loss resulting from ear malformation.

However, the concession of the Baha® system and other types of bone-anchored prostheses by the Brazilian Unified Health System only occurred in 2014 through Ordinance GM/MS 2776. Individuals with bilateral conductive or mixed hearing losses, resulting from congenital malformations, in which the use of hearing aids is not possible, can be contemplated with this technology. The indication may be unilateral or bilateral, as long as the symmetry between the thresholds obtained by bone conduction is observed^(9)^.

The indication process and the evaluation of the results obtained with transcutaneous and percutaneous systems are widely discussed in the international literature^(2,4,8,10-12)^ by demonstrating that both are effective in the auditory rehabilitation of these individuals. Currently, it is known that the advantage of the transcutaneous system is that it allows the skin to remain intact, which minimizes the post-surgical problems observed in the percutaneous, such as irritation, inflammation and infection of the skin, and even the loss of the implant^(2,13,14)^. On the other hand, the attenuation caused by the skin may favor limited gain at high frequencies in the transcutaneous system^(15-18)^.

However, few studies have aimed to compare the Baha® Attract and Baha® Connect systems^(19,20)^. The observed results indicated improvement in audibility through the analysis of pre and post adaptation free field thresholds, as well as in the speech recognition threshold (SRT), in both systems. However, in the analysis between the systems, a better performance was observed, with a significant difference in the 4kHz threshold and in the SRT for the percutaneous system^(19,20)^.

As a result, it is still necessary to investigate the correlation of post-fitting audiological findings and the speech perception of individuals when considering the importance of high frequencies in auditory recognition, especially in difficult listening situations, as in the case of competitive noise. The adaptation process and the results obtained with transcutaneous and percutaneous systems are increasingly discussed in the literature; whose studies address populations in different countries, however, there is a scarcity of data in Brazil, where there is a specific population profile with possible cultural influences on the adaptation of the BAHA.

Given the above, in order to contribute to the development of clinical protocols in the area, the objective of the present study was to longitudinally verify the influence of auditory tonal thresholds obtained in free field with transcutaneous and percutaneous BAHAs on speech perception, in conditions of silence and noise, in individuals with external and/or middle ear malformation and chronic otitis media.

## METHODS

The present is an observational, retrospective and longitudinal study, approved by the Research Ethics Committee of Hospital de Reabilitação de Anomalias Craniofaciais - University of São Paulo/Brazil, under report 3.490.345. Data collection was carried out through document analysis of medical records of patients enrolled in the Institution, with the Free and Informed Consent Form being waived. The service has a standardized clinical protocol for the evaluation and follow-up of candidates and users of the BAHA. Secondary data were collected using the Tasy hospital management software system from August to September 2019.

The eligibility criteria were individuals with bilateral conductive or mixed hearing loss who underwent surgery for unilateral implantation of the Baha® transcutaneous (Baha® Attract) or percutaneous (Baha® Connect) systems with the same processor, regardless of age and gender.

The exclusion criteria were the absence of information regarding tonal audiometry in free field and the evaluation of speech perception in some of the stages analyzed, pre-surgery and post-surgery.

The collected information was: tonal audiometry performed in free field with the research of tonal thresholds with and without the use of the prosthesis for the frequencies of 0.5, 1, 2, 3 and 4kHz, obtained with the warble modulated tone; and assessment of speech perception - sentence recognition threshold in situations of silence and noise (signal/noise ratio), obtained through the use of lists of recorded sentences, proposed by Costa^(21)^. The procedures were performed in an acoustic booth, using a two-channel audiometer model Astera 2 Madsen (Otometrics), with the acoustic box positioned at 0° azimuth and one meter away from the individual.

To obtain the sentence recognition threshold in silence (SRTS,) the ascending-descending technique was used^(22)^. The first sentence being presented at an intensity of 65dBHL without the processor and 40dBHL with the processor on. For each correctly repeated sentence, the intensity was decreased in 4dB steps until an error occurred, even for one word of the sentence. From that intensity, the presentation occurred in 2dB increments until the sentence was repeated correctly. From then on, the intensity was reduced again in 2dB steps, until the 10 sentences in the list were presented. The presentation intensities of all sentences were recorded throughout the test. The SRTS was calculated by averaging the presentation intensities of the sentences used from the first error detected (dBHL).

The determination of the sentence recognition threshold in noise (SRTN) was also based on the same technique and followed the same calculation, however, with the presentation of the initial sentence at an intensity of 65dBHL, and the competitive noise set at 60dBHL (signal/noise ratio +5dB initial), with and without the processor turned on. Additionally, the signal-to-noise ratio (S/N) is determined by subtracting the SRTN obtained from the intensity level of the competitive noise used during the test (60dBHL).

The referred data were collected in the preoperative stages - without using the processor, being considered the last available evaluation, and postoperatively in three moments: at activation, in the first month (post 1) and in the third month (post 2), use of the transcutaneous (Baha® Attract) or percutaneous (Baha® Connect) system.

The sample distribution was verified using the Shapiro-Wilk test. The measures of central tendency and variability considered were mean, median and standard deviation (SD). For comparative analysis between groups at each evaluation moment, the Mann-Whitney test was used when considering the thresholds in the field, and the paired t test for sentence recognition in silence and in noise. For intra-group comparison-analysis, one-factor analysis of variance was applied, followed by the Tukey's test for the analyzed variables. The significance level used was 5%.

## RESULTS

According to previously established eligibility criteria, 30 individuals who were unilateral users of the Baha® 4/Cochlear sound processor were included in the study and distributed as follows: Group 1 - 14 users of the Baha® Attract transcutaneous system, six females and eight males, with a mean age at activation of 12.7 years (minimum of 10 years and maximum of 17 years); and Group 2 - 16 users of the Baha® Connect percutaneous system, 13 females and three males, with a mean age at activation of 23.3 years (minimum of 12 years and maximum of 38 years).

The characterization of the casuistry regarding the etiology, type and degree of hearing loss for the 30 individuals analyzed, according to the system used, is presented in Table 1. In individuals with mixed hearing loss, the average of bone thresholds from 0.5 to 4kHz ranged from 11.25 to 39dBHL.

**Table 1:** Characterization of the casuistry regarding the etiology, type and degree of hearing loss for the 30 individuals analyzed according to the system used, transcutaneous and percutaneous

System	n	Etiology	n	Type and degree of hearing loss
Transcutaneous	14	external/middle ear malformation	7	bilateral moderate conductive
			3	bilateral severe conductive
			1	moderate conductive RE and severe LE
			3	conductive severe RE and moderate LE
Percutaneous	13	external/middle ear malformation	2	bilateral moderate conductive
			3	bilateral severe conductive
			2	conductive severe RE and moderate LE
			4	bilateral severe mixed
			1	mixed severe RE and moderate LE
			1	bilateral deep mixed
	3	chronic otitis media	2	mixed moderate RE and profound LE
			1	bilateral deep mixed

Caption: n = individuals number; RE = right ear; LE = left ear

**Figure 1:**
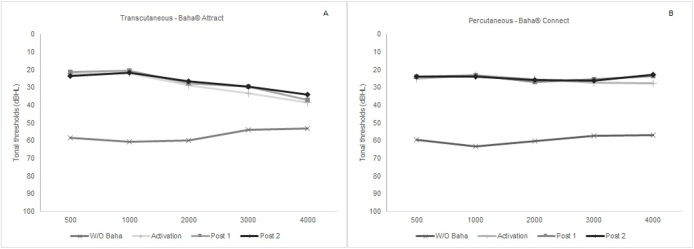
Mean of pure-field thresholds (dBHL) obtained at frequencies from 0.5 to 4kHz with and without the bone-anchored hearing aid, transcutaneous (A) and percutaneous (B) system, at activation, post 1 and post 2

Figure 1 shows the mean free-field tonal thresholds (dBHL) obtained at frequencies from 0.5 to 4kHz without and with the transcutaneous (A) and percutaneous (B) system, in the three evaluation moments: activation, post 1 and post 2.

Caption: Baha = bone-anchored hearing aid; W/O = without; dBHL = decibel level of hearing

Table 2 shows the statistical analysis when comparing the pure-field thresholds (dBHL), and speech perception with and without the transcutaneous and percutaneous system in the three evaluation moments: activation, post 1 and post 2.

**Table 2:** Statistical analysis comparing pure-field thresholds (dBHL) and speech perception with and without the bone-anchored hearing aid at activation, post 1 and post 2, for the transcutaneous and percutaneous systems

	**Tonal Threshold**		**SRTS**		**S/N**	
	**Transcut.**	**Percut**	**Transcut.**	**Percut.**	**Transcut.**	**Percut.**
W/O Baha × Activ.	<0.001*	<0.001*	<0.001*	<0.001*	<0.001*	<0.001*
Activ. × Post 1	0.357	0.906	0.938	0.993	0.271	0.991
Activ. × Post 2	0.219	0.476	0.998	0.65	0.513	0.961
Pós 1 × Post 2	0138	0.520	0.979	0.81	0.969	0.862

Simple ANOVA

* Tukey's Test (p<0.001)

Caption: SRTS = sentence recognition threshold in silence; S/N = signal to noise ratio; Transcut. = transcutaneous; Percut. = percutaneous; W/O = without; Baha = bone-anchored hearing aid; Activ. = activation

A similar analysis was performed comparing the transcutaneous and percutaneous systems (Table 3).

**Table 3:** Comparative statistical analysis between transcutaneous and percutaneous systems for pure-field thresholds (dBHL) and speech perception with and without the bone-anchored hearing aid at activation, post 1 and post 2

**Transcutaneous × Percutaneous**							
	**0.5K**	**1K**	**2K**	**3K**	**4K**	**SRTS**	**SRTN**
W/O Baha	1.000	0.465	0.937	0.187	0.200	0.411	0.672
Activation	0.531	0.936	0.111	0.012*	0,001*	0.630	0.036**
Post 1	0.178	0.060	0.121	0.001*	<0.001*	0.677	0.143
Post 2	0.982	0.196	0.192	0.006*	<0.001*	0.879	0.364

* Mann-Whitney Test, p<0.05 (tonal thresholds);

** t Test, p<0.05 (speech perception)

Caption: W/O = without; Baha = bone-anchored hearing aid; SRTS = sentence recognition threshold in silence; SRTN = signal to noise ratio

**Figure 2:**
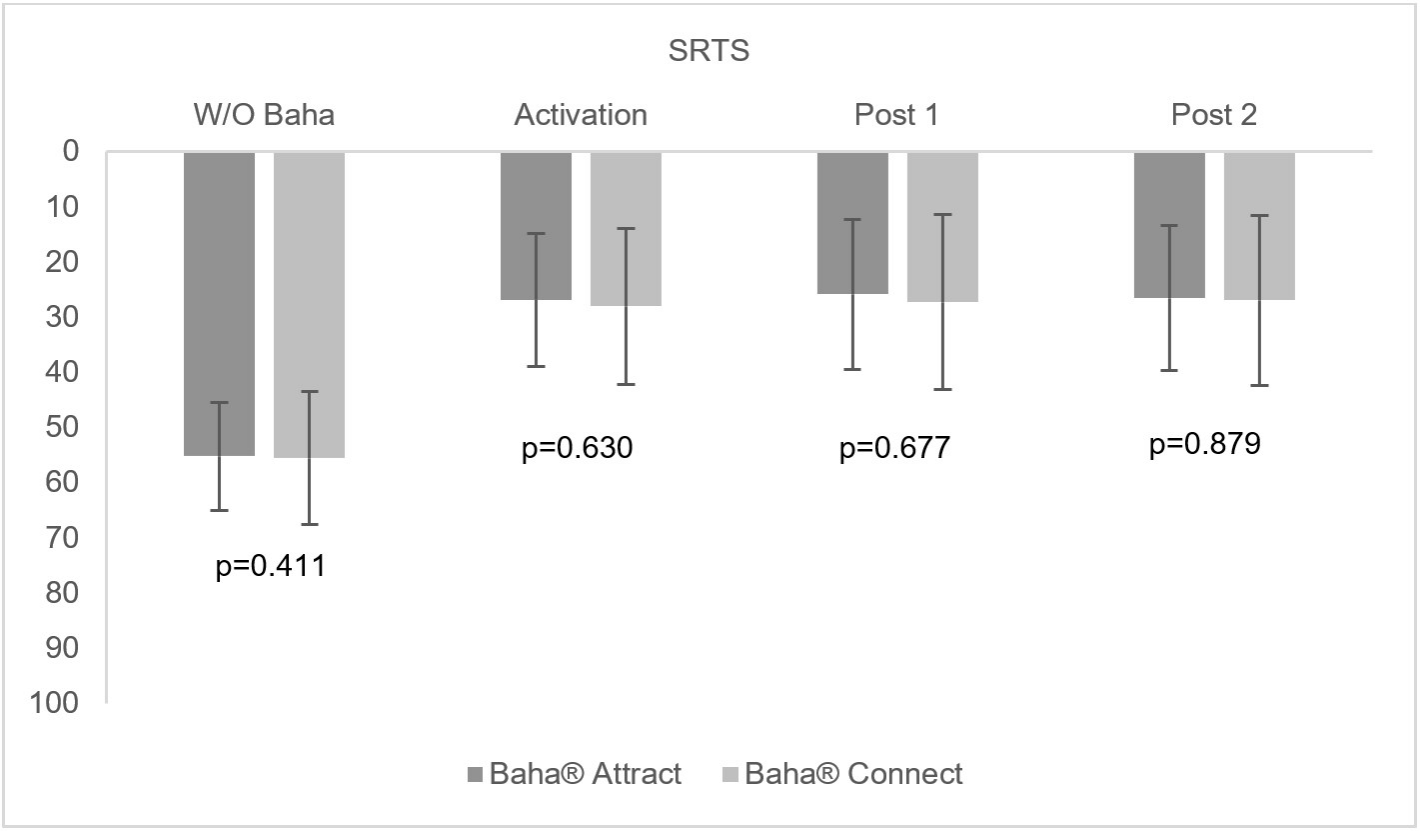
Mean sentence recognition thresholds in silence (dBHL) with and without bone-anchored hearing aids for the transcutaneous and percutaneous systems in the three evaluation moments: at activation, post 1 and post 2

**Figure 3:**
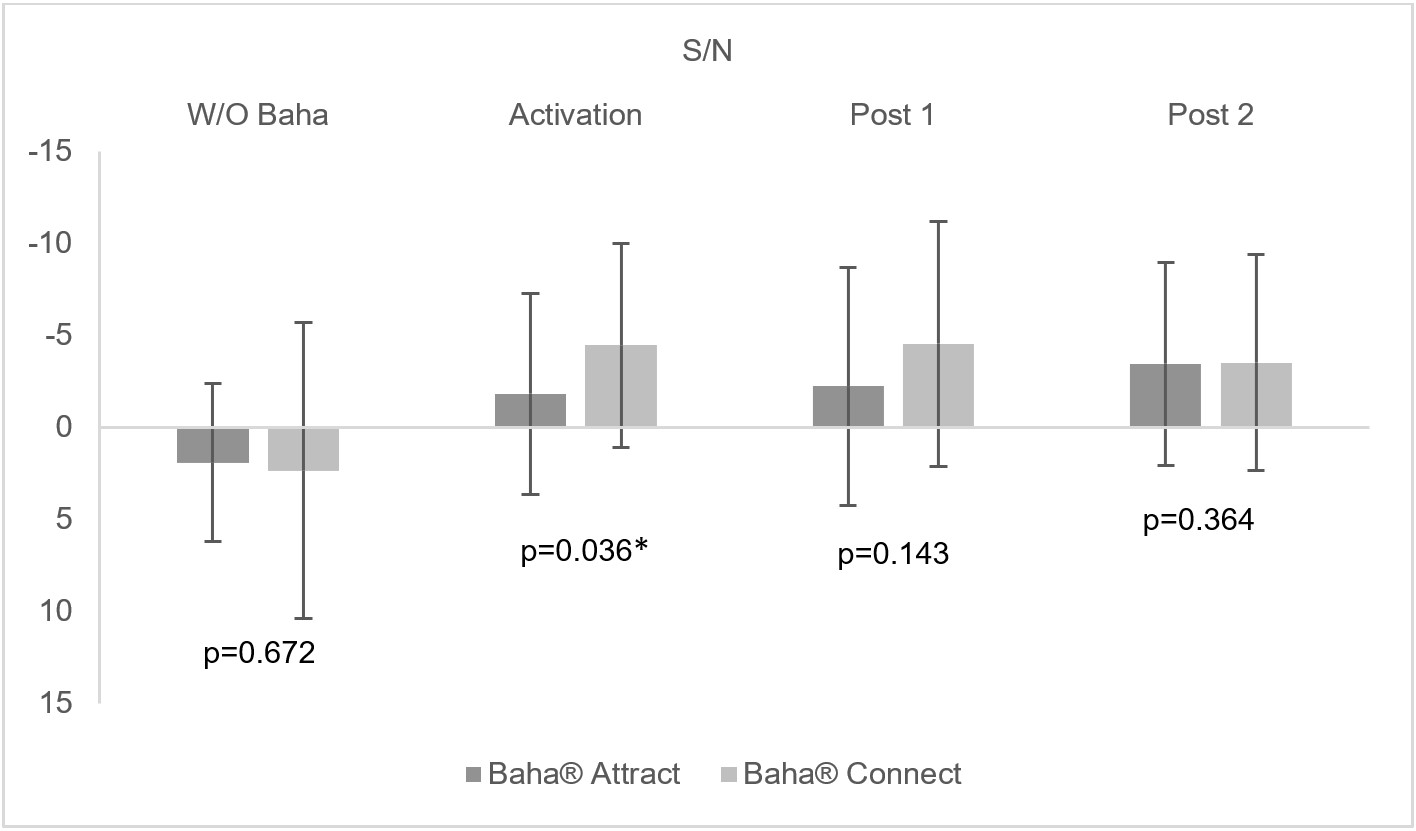
Mean S/N ratio with and without the bone-anchored hearing aid for the transcutaneous and percutaneous systems in the three evaluation moments: at activation, post 1 and post 2

Figures 2 and 3 present, respectively, the average of the sentence recognition thresholds in silence (dBHL), and the S/N ratio with and without the transcutaneous and percutaneous system in the three evaluation moments: activation, post 1 and post 2.

t Test (p>0.05)

Caption: SRTS = sentence recognition threshold in silence; W/O = without; Baha = bone-anchored hearing aid

*t Test (p<0.05)

Caption: S/N = signal to noise ratio; W/O = without; Baha = bone-anchored hearing aid

## DISCUSSION

Initially, it is important to emphasize that there was no significant difference before the surgery between the auditory thresholds in the frequencies of 0.5 to 4KHz, as well as for the performance in the recognition of sentences in silence and in noise when comparing the individuals who composed Group 1 - Baha® Attract transcutaneous system and Group 2 - Baha® Connect percutaneous system (Table 3, Figures 2 and 3).

Thus, the results obtained from 30 unilateral users of the Baha® sound processor in the transcutaneous and percutaneous systems, showed that the prosthesis provides the user with a significant improvement in audibility at all frequencies studied as soon as the device is activated (Table 2 and Figure 1). These findings show that the transcutaneous and percutaneous systems are effective in transmitting sound by bone conduction, which is necessary for auditory rehabilitation in this population^(20,23)^ corroborating with previous studies developed with children^(16,24-26)^ and adults^(14,19,20)^.

In the same Figure 1, it can be seen that the tonal thresholds obtained in the activation remained stable in the other evaluation moments since there was no significant difference when analyzing all frequencies (Table 2), for the transcutaneous and percutaneous systems. However, it was observed that in the percutaneous system, pure tone thresholds at frequencies of 3 and 4kHz are better when compared to those obtained in the transcutaneous system, a difference that was maintained in the three moments: activation, post 1 and post 2 (Table 3), despite the sensorineural component observed in individuals with chronic otitis media. Similar results were found in previous studies^(16,19,20,25)^.

In the percutaneous system, stimulation occurs directly through the pillar with consequent skull vibration, which avoids the attenuation caused by the skin observed in the transcutaneous system^(16-18)^ with greater gain for high frequencies. Specific literature describes the importance of frequencies above 3kHz for speech understanding^(10)^ especially in noisy environments^(27)^.

In the present study, as observed for the tonal thresholds, the auditory performance found in the recognition of sentences in silence, showed a significant improvement when comparing the conditions without the Baha® and the moment of activation for the two systems evaluated, transcutaneous and percutaneous, which remained stable in the other evaluation moments (Figure 2). Such results were described in previous studies, with the use of words to research the speech recognition threshold in silence^(19,20,28)^.

Although the transcutaneous system presented worse results for tonal thresholds at high frequencies, these did not impair the performance of sentence recognition in silence since there was no difference between the systems at all evaluation moments (Table 3), contrary to older studies^(16,19)^. It is interesting to observe that data from Tobia et al.^(20)^ reinforce the findings of the present study, and allow us to assume that there has been a technological improvement in sound processors that supply the impact on speech perception due to the attenuation resulting from the type of stimulation observed in the transcutaneous system for high frequencies.

Speech perception was also evaluated in a difficult listening situation, in which sentences were presented with competitive noise. As a result, a similar pattern of response to that obtained in the silent assessment was observed; that is, the improvement in the individual's performance already occurs in the activation, with a significant difference, which remains stable in the other evaluation moments for both systems (Figure 3). In the researched literature, only one study was found with analysis for the percutaneous system with similar results^(29)^. No studies were found with the selected transcutaneous system.

However, in the comparative analysis of the transcutaneous and percutaneous systems, relevant findings were obtained that may contribute to improving the criteria for indicating the Baha®. Contrary to what was observed in the silent situation, the better audibility for high frequencies found in the percutaneous group may have favored the better performance in speech recognition in noise since there was a significant difference in the S/N ratio at the moment of activation between the systems (Table 3 and Figure 3). However, over time, at the post 1 and post 2 moments, this difference ceased to be significant, that is, similar performance was observed despite the existing sensorineural component in individuals with chronic otitis media.

These findings demonstrate that the auditory stimulation provided by the continuous use of the BAHA, regardless of the system, favors speech perception abilities for situations of silence and noise and compensates for the limitation of the transcutaneous system at high frequencies.

It is important to emphasize that the Baha® adjustment for the transcutaneous and percutaneous system performed at the time of activation was maintained in the post 1 and post 2 evaluations; that is, the results obtained did not have interference from a supposed change in programming by the professional. This finding does not support the recommendation to provide greater acoustic gain for high frequencies in the transcutaneous system in order to favor the audibility of speech spectrum sounds with the aim of compensating for energy loss^(25)^.

Thus, the indication of one system or another is more related to clinical and medical aspects than to the characteristics of transcutaneous or percutaneous stimulation.

As a result, the promising prognosis in the indication of these prostheses for the individual who, due to ear malformation or chronic otitis media, cannot benefit from the conventional hearing aids is highlighted.

## CONCLUSION

The percutaneous system provided better audibility for high frequencies compared to the transcutaneous system; however, such audibility did not influence sentence recognition in the silent situation for both systems. For sentence recognition in noise, audibility for high frequencies may have provided better results in the percutaneous system on activation, however, this difference was not maintained over time, with similar performance for both systems.

## References

[1] Jardim IS, Brito R, Costa OA, Boéchat EM, Menezes PL, Couto CM, Frizzo ACF, Scharlach RC, Anastasio ART (2015). Tratado de audiologia..

[2] Fan X, Yang T, Niu X, Wang Y, Fan Y, Chen X (2019). Long-term outcomes of bone conduction hearing implants in patients with bilateral microtia-atresia. Otol Neurotol.

[3] Beutner D, Delb W, Frenzel H, Hoppe U, Hüttenbrink KB, Mlynski R (2018). Guideline implantable hearing aids: short version. HNO.

[4] (2022). Consensus statement on bone conduction devices and active middle ear implants in conductive and mixed hearing loss. Otol Neurotol.

[5] Park MJ, Lee JR, Yang CJ, Yoo MH, Jin IS, Choi CH (2016). Amplification of transcutaneous and percutaneous bone-conduction devices with a test-band in an induced model of conductive hearing loss. Int J Audiol.

[6] Röösli C, Dobrev I, Pfiffner F (2022). Transcranial attenuation in bone conduction stimulation. Hear Res.

[7] Reinfeldt S, Håkansson B, Taghavi H, Eeg-Olofsson M (2015). New developments in bone-conduction hearing implants: a review. Med Devices.

[8] Gawęcki W, Stieler OM, Balcerowiak A, Komar D, Gibasiewicz R, Karlik M (2016). Surgical, functional and audiological evaluation of new Baha(®) Attract system implantations. Eur Arch Otorhinolaryngol.

[9] Brasil (2014). Portaria GM/MS nº 2776, de 18 de dezembro de 2014. Diretrizes gerais para a atenção especializada às pessoas com deficiência auditiva no Sistema Único de Saúde (SUS).

[10] den Besten CA, Monksfield P, Bosman A, Skarzynski PH, Green K, Runge C (2019). Audiological and clinical outcomes of a transcutaneous bone conduction hearing implant: six-month results from a multicentre study. Clin Otolaryngol.

[11] Scotta G, Allam A, Dimitriadis PA, Wright K, Yardley M, Ray J (2020). Surgical and functional outcomes of two types of transcutaneous bone conduction implants. J Laryngol Otol.

[12] Rahne T, Plontke SK (2022). Systematic and audiological indication criteria for bone conduction devices and active middle ear implants. Hear Res.

[13] Fritz CG, Bojrab DI, Lin KF, Schutt CA, Babu SC, Hong RS (2020). Surgical explantation of bone-anchored hearing devices: a 10-year single institution review. Otolaryngol Head Neck Surg.

[14] Kruyt IJ, Monksfield P, Skarzynski PH, Green K, Runge C, Bosman A (2020). Results of a 2-year prospective multicenter study evaluating long-term audiological and clinical outcomes of a transcutaneous implant for bone conduction hearing. Otol Neurotol.

[15] Verstraeten N, Zarowski AJ, Somers T, Riff D, Offeciers EF (2009). Comparison of the audiologic results obtained with the bone-anchored hearing aid attached to the headband, the testband, and to the “snap” abutment. Otol Neurotol.

[16] Hol MKS, Nelissen RC, Agterberg MJH, Cremers CWRJ, Snik AFM (2013). Comparison between a new implantable transcutaneous bone conductor and percutaneous bone-conduction hearing implant. Otol Neurotol.

[17] Ellsperman SE, Nairn EM, Stucken EZ (2021). Review of bone conduction hearing devices. Audiology Res.

[18] Ontario Health (Quality) (2020). Implantable devices for single-sided deafness and conductive or mixed hearing loss: a health technology assessment. Ont Health Technol Assess Ser.

[19] Iseri M, Orhan KS, Tuncer U, Kara A, Durgut M, Guldiken Y (2015). Transcutaneous bone-anchored hearing aids versus percutaneous ones: multicenter comparative clinical study. Otol Neurotol.

[20] Tobia A, Yehudai N, Khnifes R, Shpak T, Roth O, Khayr R (2021). Hearing outcomes with percutaneous and transcutaneous BAHA® technology in conductive and mixed hearing loss. Otol Neurotol.

[21] Costa MJ (1998). Lista de sentenças em português: apresentação e estratégias de aplicação na audiologia..

[22] Levitt H, Rabiner LR (1967). Binaural release from masking for speech and gain in intelligibility. J Acoust Soc Am.

[23] Rigato C, Reinfeldt S, Håkansson B, Jansson KJ, Hol MK, Eeg-Olofsson M (2016). Audiometric comparison between the first patients with the transcutaneous bone conduction implant and matched percutaneous bone anchored hearing device users. Otol Neurotol.

[24] Lippmann E, Pritchett C, Ittner C, Hoff SR (2018). Transcutaneous osseointegrated implants for pediatric patients with aural atresia. JAMA Otolaryngol Head Neck Surg.

[25] Vaughan-Christensen L, Reed K, Smith-Olinde L (2019). Audiological outcomes utilizing a transcutaneous ossseointegrated implant system in pediatric patients. J Otolaryngol ENT Res..

[26] Oberlies NR, Castaño JE, Freiser ME, McCoy JL, Shaffer AD, Jabbour N (2020). Outcomes of BAHA connect vs BAHA attract in pediatric patients. Int J Pediatr Otorhinolaryngol.

[27] Moore BC (2016). A review of the perceptual effects of hearing loss for frequencies above 3 kHz. Int J Audiol.

[28] Rahim SA, Goh BS, Zainor S, Rahman RA, Abdullah A (2018). Outcomes of bone anchored hearing aid implant at Universiti Kebangsaan Malaysia Medical Centre (UKMMC). Indian J Otolaryngol Head Neck Surg.

[29] Catalani B, Sassi TSS, Bucuvic EC, Lourençone LFM, Alvarenga KF, Brito RV (2021). Prótese auditiva ancorada ao osso percutânea: benefícios auditivos. Audiol Commun Res.

