# Supplementary material for: Hearing rehabilitation with Baha® transcutaneous and percutaneous systems
Source: Codas. 2023 Oct 23;36(1):e20220271. doi: 10.1590/2317-1782/20232022271en (PMC10715577; doi:10.1590/2317-1782/20232022271en)
Supplement: Translation

# Translation: Reabilitação auditiva com sistemas Baha^®^ transcutâneo e percutâneo

**DOI:** 10.1590/2317-1782/20232022271pt

**Authors:** Eliane Aparecida Techi Castiquini, Kátia de Freitas Alvarenga, Lucilena Miranda de Souza, Valdéia Vieira de Oliveira, Juliana Nogueira Chaves, Luiz Fernando Manzoni Lourençone, Rubens Vuono de Brito

**Affiliations:** 1 Divisão de Saúde Auditiva, Hospital de Reabilitação de Anomalias Craniofaciais - HRAC, Universidade de São Paulo - USP - Bauru (SP), Brasil.; 2 Departamento de Fonoaudiologia, Faculdade de Odontologia de Bauru - FOB, Universidade de São Paulo - USP - Bauru (SP), Brasil.; 3 Programa de Residência Multiprofissional em Saúde Auditiva, Hospital de Reabilitação de Anomalias Craniofaciais - HRAC, Universidade de São Paulo - USP - Bauru (SP), Brasil.; 4 Hospital de Reabilitação de Anomalias Craniofaciais - HRAC, Universidade de São Paulo - USP - Bauru (SP), Brasil.; 5 Curso de Medicina, Faculdade de Odontologia de Bauru - FOB, Universidade de São Paulo - USP - Bauru (SP), Brasil.

**Keywords:** Condução Óssea, Malformação de Orelha, Audiometria da Fala, Perda Auditiva, Auxiliares de Audição

## Abstract

**Objetivo:**

Verificar longitudinalmente a influência dos limiares tonais auditivos obtidos com as próteses auditivas ancoradas no osso transcutâneas e percutâneas na percepção da fala em indivíduos com malformação de orelha externa e/ou média e Otite Média Crônica.

**Método:**

Estudo observacional, retrospectivo, de seguimento longitudinal de 30 indivíduos usuários unilaterais de sistema Baha® transcutâneo e percutâneo, para coleta de dados secundários dos limiares tonais obtidos por meio da audiometria em campo livre e do limiar de reconhecimento de sentenças no silêncio e no ruído nas condições: sem a prótese; no momento de ativação; no primeiro mês de uso (pós 1); e no terceiro mês (pós 2).

**Resultados:**

Houve diferença significante entre os limiares tonais obtidos nas frequências de 3 e 4kHz, com melhores resultados para o percutâneo em todos os momentos de avaliação. Para os dois sistemas, observou-se melhor desempenho no reconhecimento de sentenças no silêncio e ruído, com diferença significante na ativação (p<0,001), porém manteve-se estável nos demais momentos de avaliação. O sistema percutâneo mostrou melhor benefício no reconhecimento de sentenças no ruído apenas na ativação (p=0,036), quando comparado ao transcutâneo.

**Conclusão:**

O sistema percutâneo possibilitou melhor audibilidade para as frequências altas; contudo, tal audibilidade não influenciou no reconhecimento de sentenças na situação de silêncio para ambos os sistemas. Para a situação de ruído, melhores respostas foram observadas no sistema percutâneo; porém, a diferença não se manteve no decorrer do tempo.

## INTRODUÇÃO

No contexto em que não é possível adaptar aparelhos de amplificação sonora individuais (AASI) por condução aérea devido a fatores anatômicos, fisiológicos ou patológicos, como as malformações e infecções de orelha externa e/ou média recorrentes ou processos alérgicos relacionados ao molde auricular, as próteses auditivas ancoradas no osso (PAAO) viabilizam o processo de reabilitação auditiva^(1,2)^. Este tipo de dispositivo eletrônico por condução óssea proporciona a audibilidade e a percepção da fala necessárias para a aquisição e o desenvolvimento da linguagem oral, além de contribuir com o desenvolvimento escolar, com a inserção do indivíduo no mercado de trabalho e favorecer a socialização, com consequentes impactos na qualidade de vida.

A indicação da PAAO baseia-se em critérios audiológicos e médicos, como limiares auditivos de condução óssea e espessura da calota craniana, respectivamente^(3,4)^. As necessidades individuais como preocupação estética, destreza manual e expectativa aos benefícios sociais e profissionais também devem ser consideradas. Neste sentido, o indivíduo deve ser encorajado e estimulado a participar ativamente do processo de indicação, para uma tomada de decisão assertiva^(4)^.

As PAAO dispensam a inserção de moldes, olivas ou qualquer material condutor de som no meato acústico externo e possuem dois componentes: o externo (processador de som) e o interno (implantável), sendo classificadas em transcutâneas e percutâneas. Em ambas, o som é transmitido como sinal mecânico, por meio da vibração do crânio, diretamente para a cóclea^(4,5)^, o que resulta na propagação das ondas ao longo da membrana basilar e estimulação do nervo auditivo^(6)^.

A PAAO da empresa Cochlear, sistema Baha®, foi desenvolvida por Tjellstrom em 1977, utilizando o sistema Branemark de implantação^(7)^. No sistema percutâneo (Baha® Connect), o processador de som é acoplado ao osso do crânio por meio de um *abutment* (pilar) e de um parafuso de titânio, não havendo qualquer tipo de barreira entre os dois componentes. O sistema transcutâneo (Baha® Attract), por sua vez, possui, além do pino de titânio inserido ao osso, um ímã interno por meio do qual o processador é acoplado ao ímã externo que permanecerá em contato direto com a pele^(4,8)^. Importante destacar que o mesmo modelo de processador de som é usado em ambos os sistemas.

No Brasil, as primeiras cirurgias com o sistema Baha® foram realizadas no Hospital de Reabilitação de Anomalias Craniofaciais - Universidade de São Paulo, na década de 90, por meio de projeto de pesquisa voltado aos indivíduos sindrômicos com perda auditiva condutiva e mista decorrentes de malformação de orelha.

Contudo, a concessão do sistema Baha® e demais tipos de prótese ancorada no osso pelo Sistema Único de Saúde Brasileiro ocorreu apenas no ano de 2014, por meio da Portaria GM/MS 2776. Indivíduos com perdas auditivas bilaterais condutivas ou mistas, decorrentes de malformações congênitas, nas quais o uso do AASI não é possível, podem ser contemplados com essa tecnologia. A indicação pode ser uni ou bilateral, desde que seja observada a simetria entre os limiares obtidos por condução óssea^(9)^.

O processo de indicação e a avaliação dos resultados obtidos com as PAAO transcutânea e percutânea são amplamente discutidos na literatura internacional^(2,4,8,10-12)^, ao demonstrarem que ambas são eficazes na reabilitação auditiva destes indivíduos. Atualmente, sabe-se que a vantagem do sistema transcutâneo é a de permitir que a pele permaneça intacta, o que minimiza os problemas pós-cirúrgicos observados no percutâneo, como irritação, inflamação e infecção da pele e, até mesmo, a perda do implante^(2,13,14)^. Por outro lado, a atenuação provocada pela pele pode favorecer ganho limitado nas frequências altas no sistema transcutâneo^(15-18)^.

Entretanto, poucos estudos tiveram como objetivo a comparação entre os sistemas Baha® Attract e Baha® Connect^(19,20)^. Os resultados observados indicaram melhora na audibilidade por meio da análise dos limiares em campo livre pré e pós adaptação, assim como no limiar de reconhecimento da fala (LRF) em ambos os sistemas. Porém, na análise entre os sistemas, foi constatado desempenho melhor, com diferença significante, no limiar de 4kHz e no LRF para o sistema percutâneo^(19,20)^.

Neste sentido, ainda se faz necessário investigar a correlação dos achados audiológicos pós adaptação e a percepção da fala dos indivíduos ao considerar a importância das frequências altas no reconhecimento auditivo, principalmente em situação de escuta difícil, como no caso do ruído competitivo. O processo de adaptação e os resultados obtidos com os sistemas transcutâneos e percutâneos são cada vez mais discutidos na literatura; cujos estudos abordam populações em diversos países; contudo, há escassez de dados no Brasil, onde há um perfil específico de população com possíveis influências culturais na adaptação das PAAO.

Diante do exposto, a fim de contribuir na elaboração de protocolos clínicos na área, o objetivo deste estudo foi verificar longitudinalmente a influência dos limiares tonais auditivos obtidos em campo livre com as PAAO transcutâneas e percutâneas na percepção da fala, nas condições de silêncio e ruído, em indivíduos com malformação de orelha externa e/ou média e Otite Média Crônica.

## MÉTODO

Trata-se de um estudo observacional, retrospectivo e longitudinal, aprovado pelo Comitê de Ética em Pesquisa do Hospital de Reabilitação de Anomalias Craniofaciais - Universidade de São Paulo, sob parecer 3.490.345. A coleta de dados foi realizada por meio da análise documental de prontuários de pacientes matriculados na Instituição, sendo dispensado o Termo de Consentimento Livre e Esclarecido. O serviço possui protocolo clínico padronizado para a avaliação e o acompanhamento de candidatos e usuários de PAAO. Os dados secundários foram coletados por meio do sistema de *software* de gestão hospitalar Tasy, no período de agosto a setembro de 2019.

Os critérios de elegibilidade foram: indivíduos com perda auditiva condutiva ou mista bilateral que realizaram cirurgia para implantação unilateral dos sistemas Baha® transcutâneo (Baha® Attract) ou percutâneo (Baha® Connect) com o mesmo processador, independentemente da idade e gênero.

Os critérios de exclusão foram a ausência de informações referentes à audiometria tonal em campo livre e a avaliação da percepção da fala em algumas das etapas analisadas, pré-cirúrgica e pós cirúrgica.

As informações coletadas foram: audiometria tonal realizada em campo livre com a pesquisa dos limiares tonais sem e com o uso da prótese para as frequências de 0,5, 1, 2, 3 e 4kHz, obtidos com o tom modulado w*arble*; e avaliação da percepção da fala - limiar de reconhecimento de sentenças nas situações de silêncio e ruído (relação sinal/ruído), obtida por meio da utilização das listas de sentenças gravadas, propostas por Costa^(21)^. Os procedimentos foram realizados em cabina acústica, utilizando-se um audiômetro de dois canais modelo Astera 2 Madsen (Otometrics), com a caixa acústica posicionada a 0° azimute a um metro de distância do indivíduo.

Para a obtenção do limiar de reconhecimento de sentenças no silêncio (LRSS) foi utilizada a técnica ascendente-descendente^(22)^, sendo a primeira sentença apresentada na intensidade de 65dBNA sem o processador e 40dBNA com processador ligado. A cada sentença repetida corretamente, a intensidade foi diminuída em passos de 4dB até a ocorrência de erro, mesmo que em uma palavra da sentença. A partir dessa intensidade, a apresentação ocorreu em aumentos de 2dB até que a sentença fosse repetida corretamente. A partir de então, a intensidade foi reduzida novamente em passos de 2dB, até que as 10 sentenças da lista fossem apresentadas. As intensidades de apresentação de todas as sentenças foram anotadas durante todo o teste. O LRSS foi calculado pela média das intensidades de apresentação das sentenças a partir do primeiro erro constatado (dBNA).

A determinação do limiar de reconhecimento de sentenças no ruído (LRSR) também foi baseada na mesma técnica e seguindo o mesmo cálculo, porém, com a apresentação da sentença inicial na intensidade de 65dBNA e o ruído competitivo fixado em 60dBNA (relação sinal/ruído inicial de +5dB), sem e com o processador ligado. Adicionalmente, a relação sinal/ruído (S/R) é determinada subtraindo o LRSR obtido do nível de intensidade do ruído competitivo utilizado durante o teste (60dBNA).

Os referidos dados foram coletados nas etapas pré-operatória - sem o uso do processador, sendo considerada a última avaliação disponível, e pós-operatória em três momentos: na ativação, no primeiro mês (pós 1) e no terceiro mês (pós 2) de uso do sistema transcutâneo (Baha® Attract) ou percutâneo (Baha® Connect).

A distribuição da amostra foi verificada por meio do teste Shapiro-Wilk. As medidas de tendência central e de variabilidade consideradas foram: média, mediana e desvio padrão (DP). Para análise comparativa entre grupos em cada momento de avaliação foi utilizado o teste Mann-Whitney ao considerar os limiares em campo e o teste t pareado para o reconhecimento de sentenças no silêncio e no ruído. Para a comparação intra-grupos, aplicou-se a análise de variância de um fator e, posteriormente, o Teste Tukey para as variáveis analisadas. O nível de significância utilizado foi de 5%.

## RESULTADOS

Conforme critérios de elegibilidade previamente estabelecidos, 30 indivíduos usuários unilaterais do processador de som Baha® 4/Cochlear foram incluídos no estudo e distribuídos da seguinte forma: Grupo 1 - 14 usuários do sistema transcutâneo Baha® Attract, sendo seis do gênero feminino e oito do gênero masculino, com média de idade na ativação de 12,7 anos (mínima de 10 anos e máxima de 17 anos); e Grupo 2 - 16 usuários do sistema percutâneo Baha® Connect, sendo 13 do gênero feminino e três do gênero masculino, com média de idade na ativação de 23,3 anos (mínima de 12 anos e máxima de 38 anos).

A caracterização da casuística quanto à etiologia, tipo e grau da perda auditiva para os 30 indivíduos analisados, de acordo com o sistema utilizado, está apresentada na Tabela 1. Nos indivíduos com perda auditiva mista, a média de limiares ósseos de 0,5 a 4kHz variou de 11,25 a 39dBNA.

**Tabela 1:** Caracterização da casuística quanto à etiologia, tipo e grau de perda auditiva, para os 30 indivíduos analisados de acordo com o sistema utilizado, transcutâneo e percutâneo

Sistema	N	Etiologia	n	Tipo e grau da perda auditiva
Transcutâneo	14	malformação de orelha externa/média	7	condutiva moderada bilateral
			3	condutiva severa bilateral
			1	condutiva moderada OD e severa OE
			3	condutiva severa OD e moderada OE
Percutâneo	13	malformação de orelha externa/média	2	condutiva moderada bilateral
			3	condutiva severa bilateral
			2	condutiva severa OD e moderada OE
			4	mista severa bilateral
			1	mista severa OD e moderada OE
			1	mista profunda bilateral
	3	otite média crônica	2	mista moderada OD e profunda OE
			1	mista profunda bilateral

Legenda: n = número de indivíduos; OD = orelha direita; OE = orelha esquerda

A Figura 1 apresenta a média dos limiares tonais em campo livre (dBNA) obtidos nas frequências de 0,5 a 4kHz sem e com a PAAO sistema transcutâneo (A) e percutâneo (B), nos três momentos de avaliação: ativação, pós 1 e pós 2.

**Figura 1:** Média dos limiares tonais em campo livre (dBNA) obtidos nas frequências de 0,5 a 4kHz sem e com a prótese auditiva ancorada no osso, sistema transcutâneo (A) e percutâneo (B), na ativação, pós 1 e pós 2

Legenda: Baha = bone-anchored hearing aid; dBNA = decibel nível de audição

Na Tabela 2, encontra-se a análise estatística ao comparar os limiares tonais em campo livre (dBNA) e percepção da fala sem e com a PAAO sistema transcutâneo e percutâneo nos três momentos de avaliação: ativação, pós 1 e pós 2.

**Tabela 2:** Análise estatística ao comparar os limiares tonais em campo livre (dBNA) e percepção da fala sem e com a prótese auditiva ancorada no osso na ativação, pós 1 e pós 2, para os sistemas transcutâneo e percutâneo

	**Limiar auditivo**		**LRSS**		**S/R**	
	**Transcutâneo**	**Percutâneo**	**Transcutâneo**	**Percutâneo**	**Transcutâneo**	**Percutâneo**
Sem PAAO x Ativ.	<0,001*	<0,001*	<0,001*	<0,001*	<0,001*	<0,001*
Ativ. × Pós 1	0,357	0,906	0,938	0,993	0,271	0,991
Ativ. × Pós 2	0,219	0,476	0,998	0,65	0,513	0,961
Pós 1 × Pós 2	0138	0,520	0,979	0,81	0,969	0,862

ANOVA simples

* Teste Tukey (p<0,001)

Legenda: PAAO = prótese auditiva ancorada no osso; Ativ. = ativação; LRSS = limiar de reconhecimento de sentenças no silêncio; S/R = relação sinal-ruído

Semelhante análise foi realizada comparando-se os sistemas transcutâneo e percutâneo (Tabela 3).

**Tabela 3:** Análise estatística comparativa entre sistema transcutâneo e percutâneo para os limiares tonais em campo livre (dBNA) e percepção da fala sem e com a prótese auditiva ancorada no osso na ativação, pós 1 e pós 2

**Transcutâneo** × **Percutâneo**							
	**0,5K**	**1K**	**2K**	**3K**	**4K**	**LRSS**	**LRSR**
Sem PAAO	1,000	0,465	0,937	0,187	0,200	0,411	0,672
Ativação	0,531	0,936	0,111	0,012*	0,001*	0,630	0,036**
Pós 1	0,178	0,060	0,121	0,001*	<0,001*	0,677	0,143
Pós 2	0,982	0,196	0,192	0,006*	<0,001*	0,879	0,364

* Teste Mann-Whitney, p<0,05 (limiares tonais);

** Teste t, p<0,05 (percepção da fala)

Legenda: PAAO = prótese auditiva ancorada no osso; LRSS = limiar de reconhecimento de sentenças no silêncio; LRSR = limiar de reconhecimento de sentenças no ruído

As Figuras 2 e 3 apresentam, respectivamente, a média dos limiares de reconhecimento de sentenças no silêncio (dBNA) e da relação S/R sem e com a PAAO sistema transcutâneo e percutâneo nos três momentos de avaliação: ativação, pós 1 e pós 2.

**Figura 2:** Média dos limiares de reconhecimento de sentenças no silêncio (dBNA) sem e com a prótese auditiva ancorada no osso, para os sistemas transcutâneo e percutâneo, nos três momentos de avaliação: na ativação, pós 1 e pós 2

Teste t (p>0,05)

Legenda: PAAO = prótese auditiva ancorada no osso; LRSS = limiar de reconhecimento de sentenças no silêncio

**Figura 3:** Média da relação S/R sem e com a prótese auditiva ancorada no osso, para os sistemas transcutâneo e percutâneo, nos três momentos de avaliação: na ativação, pós 1 e pós 2

*Teste t (p<0,05)

Legenda: PAAO = prótese auditiva ancorada no osso; S/R = relação sinal-ruído

## DISCUSSÃO

Inicialmente, é importante ressaltar que não houve diferença significante previamente à cirurgia entre os limiares auditivos nas frequências de 0,5 a 4 KHz, assim como no reconhecimento de sentenças no silêncio e no ruído, quando comparados os indivíduos que compuseram o Grupo 1 - sistema transcutâneo Baha® Attract e Grupo 2 - sistema percutâneo Baha® Connect (Tabela 3, Figuras 2 e 3).

Assim, os resultados obtidos em 30 indivíduos usuários unilaterais do processador de som Baha®, nos sistemas transcutâneo e percutâneo, mostraram que as PAAO proporcionam ao usuário melhora significante na audibilidade em todas as frequências pesquisadas, já na ativação do dispositivo (Tabela 2 e Figura 1). Estes achados evidenciam que os sistemas transcutâneo e percutâneo são eficazes em transmitir o som por condução óssea, necessária para a reabilitação auditiva nesta população^(20,23)^, corroborando com estudos anteriores desenvolvidos com crianças^(16,24-26)^ e adultos^(14,19,20)^.

Na mesma Figura 1, constata-se que os limiares tonais obtidos na ativação se mantiveram estáveis nos demais momentos de avaliação, pois não houve diferença significante quando analisadas todas as frequências (Tabela 2), para os sistemas transcutâneo e percutâneo. Entretanto, foi observado que no sistema percutâneo os limiares tonais nas frequências de 3 e 4kHz são melhores quando comparados com os obtidos no sistema transcutâneo, diferença que se manteve nos três momentos: ativação, pós 1 e pós 2 (Tabela 3), apesar do componente sensorioneural observado nos indivíduos com Otite Média Crônica. Resultados semelhantes foram encontrados em estudos anteriores^(16,19,20,25)^.

No sistema percutâneo, a estimulação ocorre diretamente pelo pilar com consequente vibração do crânio, o que evita a atenuação provocada pela pele observada no sistema transcutâneo^(16-18)^, com maior ganho para as frequências altas. A literatura específica descreve a importância das frequências acima de 3kHz para a compreensão da fala^(10)^,principalmente em ambientes ruidosos^(27)^.

Neste estudo, assim como observado para os limiares tonais, o desempenho auditivo encontrado no reconhecimento de sentenças no silêncio demonstrou melhora significante quando comparadas as condições sem a PAAO e o momento da ativação para os dois sistemas avaliados, transcutâneo e percutâneo, que se manteve estável nos demais momentos de avaliação (Figura 2). Tais resultados foram descritos em estudos anteriores, com o uso de palavras para pesquisa do limiar de reconhecimento de fala no silêncio^(19,20,28)^.

Apesar do sistema transcutâneo apresentar resultados piores para os limiares tonais nas frequências altas, estes não prejudicaram o desempenho de reconhecimento de sentenças no silêncio, visto que não houve diferença entre os sistemas em todos os momentos de avaliação (Tabela 3), ao contrário de estudos mais antigos^(16,19)^. Interessante observar que os dados de Tobia et al.^(20)^ reforçam os achados do presente estudo e permitem supor que houve um aprimoramento tecnológico nos processadores de som que suprem o impacto na percepção da fala decorrente da atenuação resultante do tipo de estimulação observada no sistema transcutâneo para as frequências altas.

A percepção da fala foi avaliada também em situação de escuta difícil, na qual as sentenças foram apresentadas com ruído competitivo. Como resultado, constatou-se semelhante padrão de resposta à obtida na avaliação em silêncio; ou seja, a melhora no desempenho do indivíduo já ocorre na ativação, com diferença significante, que se mantém estável nos demais momentos de avaliação para ambos os sistemas (Figura 3). Na literatura pesquisada, encontrou-se apenas um estudo com análise para o sistema percutâneo com resultados semelhantes^(29)^. Não foram encontrados estudos com o sistema transcutâneo selecionado.

Contudo, na análise comparativa dos sistemas transcutâneo e percutâneo, relevantes achados obtidos podem contribuir para aprimorar os critérios de indicação da PAAO. Ao contrário do observado na situação em silêncio, a melhor audibilidade para as frequências altas encontrada no grupo percutâneo pode ter favorecido o melhor desempenho no reconhecimento de fala no ruído, uma vez que houve diferença significante na relação S/R no momento da ativação entre os sistemas (Tabela 3 e Figura 3). Entretanto, ao longo do tempo, nos momentos pós 1 e pós 2, esta diferença deixou de ser significante, ou seja, semelhante desempenho foi observado apesar do componente sensorioneural existente nos indivíduos com Otite Média Crônica.

Estes achados demonstram que a estimulação auditiva proporcionada pelo uso contínuo da PAAO, independente do sistema, favorece as habilidades de percepção da fala para as situações de silêncio e ruído e compensa a limitação do sistema transcutâneo nas frequências altas.

Importante ressaltar que, o ajuste da PAAO para o sistema transcutâneo e percutâneo realizado no momento da ativação foi mantido nas avaliações pós 1 e pós 2; ou seja, os resultados obtidos não tiveram interferência de suposta mudança na programação pelo profissional. Este achado não sustenta a recomendação de fornecer maior ganho acústico para as frequências altas no sistema transcutâneo de modo a favorecer a audibilidade dos sons do espectro de fala, com o intuito de compensar a perda de energia^(25)^.

Assim, a indicação de um sistema ou outro está mais relacionada aos aspectos clínicos e médicos do que às características da estimulação transcutânea ou percutânea.

Ressalta-se, com isso, o prognóstico promissor na indicação dessas próteses para o indivíduo que, devido à malformação de orelha ou Otite Média Crônica, não pode se beneficiar do aparelho de amplificação sonora convencional.

## CONCLUSÃO

O sistema percutâneo possibilitou melhor audibilidade para as frequências altas em comparação ao transcutâneo; no entanto, tal audibilidade não influenciou no reconhecimento de sentenças na situação de silêncio para ambos os sistemas. Para o reconhecimento de sentenças em situação de ruído, a audibilidade para as frequências altas pode ter propiciado melhor resultado no sistema percutâneo na ativação; porém, esta diferença não se manteve no decorrer do tempo, com desempenho semelhante para ambos os sistemas.
